# Population-Level Reduction in Adult Mortality after Extension of Free Anti-Retroviral Therapy Provision into Rural Areas in Northern Malawi

**DOI:** 10.1371/journal.pone.0013499

**Published:** 2010-10-19

**Authors:** Sian Floyd, Anna Molesworth, Albert Dube, Emmanuel Banda, Andreas Jahn, Charles Mwafulirwa, Bagrey Ngwira, Keith Branson, Amelia C. Crampin, Basia Zaba, Judith R. Glynn, Neil French

**Affiliations:** 1 Department of Infectious Disease Epidemiology, London School of Hygiene and Tropical Medicine, London, United Kingdom; 2 Karonga Prevention Study, Chilumba, Karonga, Malawi; University of Cape Town, South Africa

## Abstract

**Background:**

Four studies from sub-Saharan Africa have found a substantial population-level effect of ART provision on adult mortality. It is important to see if the impact changes with time since the start of treatment scale-up, and as treatment moves to smaller clinics.

**Methods and Findings:**

During 2002-4 a demographic surveillance site (DSS) was established in Karonga district, northern Malawi. Information on births and deaths is collected monthly, with verbal autopsies conducted for all deaths; migrations are updated annually. We analysed mortality trends by comparing three time periods: pre-ART roll-out in the district (August 2002–June 2005), ART period 1 (July 2005–September 2006) when ART was available only in a town 70 km away, and ART period 2 (October 2006–September 2008), when ART was available at a clinic within the DSS area. HIV prevalence and ART uptake were estimated from a sero-survey conducted in 2007/2008. The all-cause mortality rate among 15–59 year olds was 10.2 per 1000 person-years in the pre-ART period (288 deaths/28285 person-years). It fell by 16% in ART period 1 and by 32% in ART period 2 (95% CI 18%–43%), compared with the pre-ART period. The AIDS mortality rate fell from 6.4 to 4.6 to 2.7 per 1000 person-years in the pre-ART period, period 1 and period 2 respectively (rate ratio for period 2 = 0.43, 95% CI 0.33–0.56). There was little change in non-AIDS mortality. Treatment coverage among individuals eligible to start ART was around 70% in 2008.

**Conclusions:**

ART can have a dramatic effect on mortality in a resource-constrained setting in Africa, at least in the early years of treatment provision. Our findings support the decentralised delivery of ART from peripheral health centres with unsophisticated facilities. Continued funding to maintain and further scale-up treatment provision will bring large benefits in terms of saving lives.

## Introduction

HIV/AIDS is responsible for around one half to two-thirds of deaths among 15–59 year olds in Southern and East Africa, and both HIV prevalence and incidence remain high[1].

Antiretroviral therapy (ART) can have a dramatic effect in reducing deaths due to HIV/AIDS, and the Millennium Development Goals (MDGs) include the target of universal access to treatment for all those who need it by 2010. Billions of dollars are being invested in ART programmes to make treatment widely available[2], [3]. Following rapid scale-up of treatment in the last five years[4], systematic reviews of data from cohorts in non-research settings in sub-Saharan Africa show that median retention in care is around 80% (range 55–93%) 12 months after first starting ART, and around 70% (range 64–87%) 3 years after first starting ART[5], [6], while findings published since mid-2009 are consistent with this[7], [8], [9]. Published data on survival and retention in care from national-level ART treatment programmes in Southern and East Africa are available from Malawi, Rwanda, South Africa, Botswana, and Zambia[9], [10], [11], [12], [13]. Among all of the 72666 patients who first registered for ART in the public sector in Malawi during 2004-6, 76% were retained in care 12 months after starting ART[10]. In Rwanda, retention in care was 86% at 12 months after starting ART, among patients enrolled in the national ART programme during 2004-5[11], while among the earliest patients enrolled in Botswana's government ART programme around two-thirds were retained in care at 3 years[12].

The proportion of adult mortality that can be averted by ART provision depends on HIV prevalence, the proportion of HIV-infected individuals who know their status and whose eligibility for ART is assessed, and on the proportion of eligible individuals who start and stay in treatment programmes. In particular, mortality and loss to follow-up between the time of first being identified as eligible for ART and starting treatment can be high[14], [15], [16], [17], [18], [19], [20].

To date, there are 4 published studies of the population-level effect of ART roll-out on adult mortality in sub-Saharan Africa[21], [22], [23], [24]. In Addis Ababa in Ethiopia, cause-specific mortality data collected from burial sites showed that mortality due to AIDS fell by around 50% among 20–64 year olds during the two years after ART was first provided for free[21]. In KwaZulu Natal district, South Africa, mortality due to AIDS among 25–49 year olds fell progressively during the ART roll-out years 2004-6, compared to 2002-3, with a reduction in AIDS mortality of 22% in women and 29% in men, with no reduction in non-AIDS mortality[22]. In Thyolo district in southern Malawi, a setting in which the universal access target of 80% of those estimated to be urgently in need of ART was reached in 2007, data on all deaths (child and adult) registered by traditional authorities indicate that mortality fell by 37% between 2000 and 2007[24]. In Karonga district, northern Malawi, we have previously shown early evidence of the population-level impact of ART provision on adult mortality from a demographic surveillance site (DSS): all-cause mortality in 15–59 year olds fell by around 10% during the eight months immediately following the opening of the first ART clinic in the district at the end of June 2005[23].

In Malawi, adult HIV prevalence is around 14%, meaning that around 1 million adults are HIV-infected. Implementation of the public sector ART programme began in 2004, and by the end of 2008 around 147000 people were on treatment[25], out of an estimated 200000 eligible individuals, in around 100 health facilities[10]. Since our initial study, ART has become available at additional health facilities in Karonga district, including one within the DSS area, and the number of individuals accessing ART has increased substantially. It is important to see whether the impact of ART changes with time since the start of treatment scale-up, and whether it can be sustained through small rural clinics.

In this paper, we present data from the Karonga DSS on changes in all-cause and AIDS mortality, comparing the first 3 years of ART roll-out in the district, from the beginning of July 2005 to the end of September 2008, with the pre-ART time period from mid-2002 to mid-2005. We then relate the changes to estimates of treatment need and ART uptake, and to 12-month survival and retention in care at the health facility that provides ART within the DSS area.

## Methods

### Ethics statement

Ethical approval for the study was granted by the National Health Sciences Research Committee of Malawi (NHSRC protocol number 419) and the Ethics Committee of the London School of Hygiene & Tropical Medicine (ethics committee number 5081). For monitoring of births, deaths, and in- and out-migrations in the DSS, interviews were only conducted if verbal consent was given by the household head and by the respective household members, and consent was documented by the interview sheet that was filled: the ethics committees both agreed that written consent was not required. For the HIV sero-survey, written consent was obtained from all participants.

Karonga district is a rural area in northern Malawi, and most of the population are subsistence farmers. HIV prevalence in adults was 2% in the late 1980s, 13% in the late 1990s[26] and 10–14% during 2000–2008[27]. The first-line ART regimen is a fixed-dose combination tablet of stavudine, lamivudine, and nevirapine, taken twice daily[28]. During 2005–2008, individuals were eligible for ART if they were in WHO clinical stage 3 or 4, or had a CD4 count<250 cells/mm^3^.

During the period 2002-4 a demographic surveillance site (DSS) was established in the south of the district in a population of 32000, following a baseline census[29]. Sufficient identifying information is collected so that all individuals can be traced over time. Information on births and deaths is collected monthly and information on individual in-and out-migration is updated once a year.

Verbal autopsies are conducted for all deaths, allowing AIDS and non-AIDS deaths to be distinguished[23]. The verbal autopsies are conducted by health assistants with additional training, using a semi-structured questionnaire similar to the INDEPTH tool[30] adapted from the standard World Health Organization questionnaire. Whenever possible, the informant is a close relative of the deceased who nursed them through their final illness, and the information is collected around 4–6 weeks after the death. Each verbal autopsy questionnaire is independently reviewed by 2 clinicians, and in the event of discrepancies a third reviewer makes the decision on cause of death. If a decision cannot be made on the cause of death, then it is coded as un-specifiable.

Data were double-entered using Access® and analysed using Stata 10 (Stata corp., College Station, Texas, USA). Data were available for analysis from August 2002 up to the end of September 2008, with person-time calculated from the time an individual was first resident in the DSS until the earliest of date of death, out-migration, or end of the follow-up period. If an individual left and later returned to live in the DSS area, the time they were away was not counted towards their person-time. For analysis of AIDS mortality, deaths due to non-AIDS and un-specifiable causes were censored. For analysis of non-AIDS mortality, deaths due to AIDS were censored.

Individuals aged 60+ years old were excluded from the analysis of cause-specific mortality, for two reasons. First, in the pre-ART period only around 7% of deaths in this age group were attributed to AIDS: thus, ART could have little population-level effect on mortality in this group. Second, in this age group 18% of deaths were of un-specifiable cause. Nonetheless, we present trends in all-cause mortality among individuals aged 60+ years old, for comparison with trends in younger individuals.

We defined 3 time periods:

1. The pre-ART period (August 2002- end June 2005);
2. ART period 1 (July 2005–end September 2006), when ART was available only in a town 70 km from the DSS area;
3. ART period 2 (October 2006–end September 2008), when ART was available from a clinic within the DSS area.

We also examined mortality separately in the first and second year of period two, to see if there were any trends within that period.

Malawi's north-south highway bisects the study area, with one spur road to the clinic within the area. Proximity to the tarmac road facilitates access to the ART clinics, and HIV prevalence is higher close to this road compared with more remote areas (unpublished data). Approximately half the population lives within 1 km of the road (hereafter “rural roadside”), and the rest more remotely (hereafter “rural remote”). Analyses were stratified by area of residence, age group (15–29 years, 30–44 years, 45–59 years), and sex. Poisson regression was used to calculate rate ratios (RRs) for the effect of time period, both stratified on and adjusted for age group, sex, and area of residence. In the stratified analyses, statistical evidence for whether the effect of time period varied by each of age, sex, and area of residence was obtained from a test for interaction.

Between September 2007 and October 2008 an HIV sero-survey was conducted in the DSS area, offering HIV testing to all individuals aged 15 or more years old at their own homes, using rapid tests following a parallel testing algorithm. The sero-survey included questions on whether an individual had ever taken, or was currently taking, ART. Cross-sectional analyses of these data were done to estimate age-, sex-, and area-specific uptake of ART overall, and also restricted to individuals who tested HIV-positive during the sero-survey.

Estimation of ART need was based on a Weibull regression model fitted to age-specific survival patterns of 196 HIV-positive individuals from a retrospective cohort study conducted 1998–2000[31], in the same way as previously described in a comparative analysis of African community study data[32]. The parameters from the fitted Weibull model were then applied to the individuals who tested HIV-positive during the 2007/8 sero-survey, to calculate their predicted probability of death by 3 years later, on the assumption that an individual who is within 3 years of death is in need of ART[33]. These predicted probabilities were then summed to estimate the proportion of HIV-positive individuals who were in need of ART in 2007/2008, among those who agreed to give blood for HIV-testing in the sero-survey.

From the end of January 2008, all newly registered HIV-positive patients at the ART clinic who live within the DSS area have been invited to join a cohort study. Participants are all able to access CD4 count testing. Survival analysis of these data was done to estimate 12-month retention in care after starting ART.

## Results

Since the launch of the DSS in mid-2002, to the end of September 2008, 655 deaths were observed in a total of 77179 person-years among individuals aged 15–59 years old, a crude death rate of 8.5 per 1000 person-years. Cause of death was un-specifiable for 6% (40/655) of deaths.

Initially all analysis of mortality trends was done separately for the two 12-month time periods in which there was an ART clinic in the DSS area. Mortality rates were very similar in these two time periods, so only the combined results are shown.

Among individuals aged 60+ years old, the all-cause mortality rate was little changed over the 6-year period, with mortality rates per 1000 person-years of 43.6 (149 deaths in 3414 person-years) pre-ART, 41.5 (95 deaths in 2290 person-years) during ART period 1, and 39.4 (147 deaths in 3731 person-years) during ART period 2. Corresponding rate ratios (RR), compared with the pre-ART period and adjusted for area of residence and sex, were 0.99 (95% CI 0.77–1.29) and 0.95 (95% CI 0.75–1.19) respectively.

### All-cause mortality in 15–59 year olds

**Figure 1:**
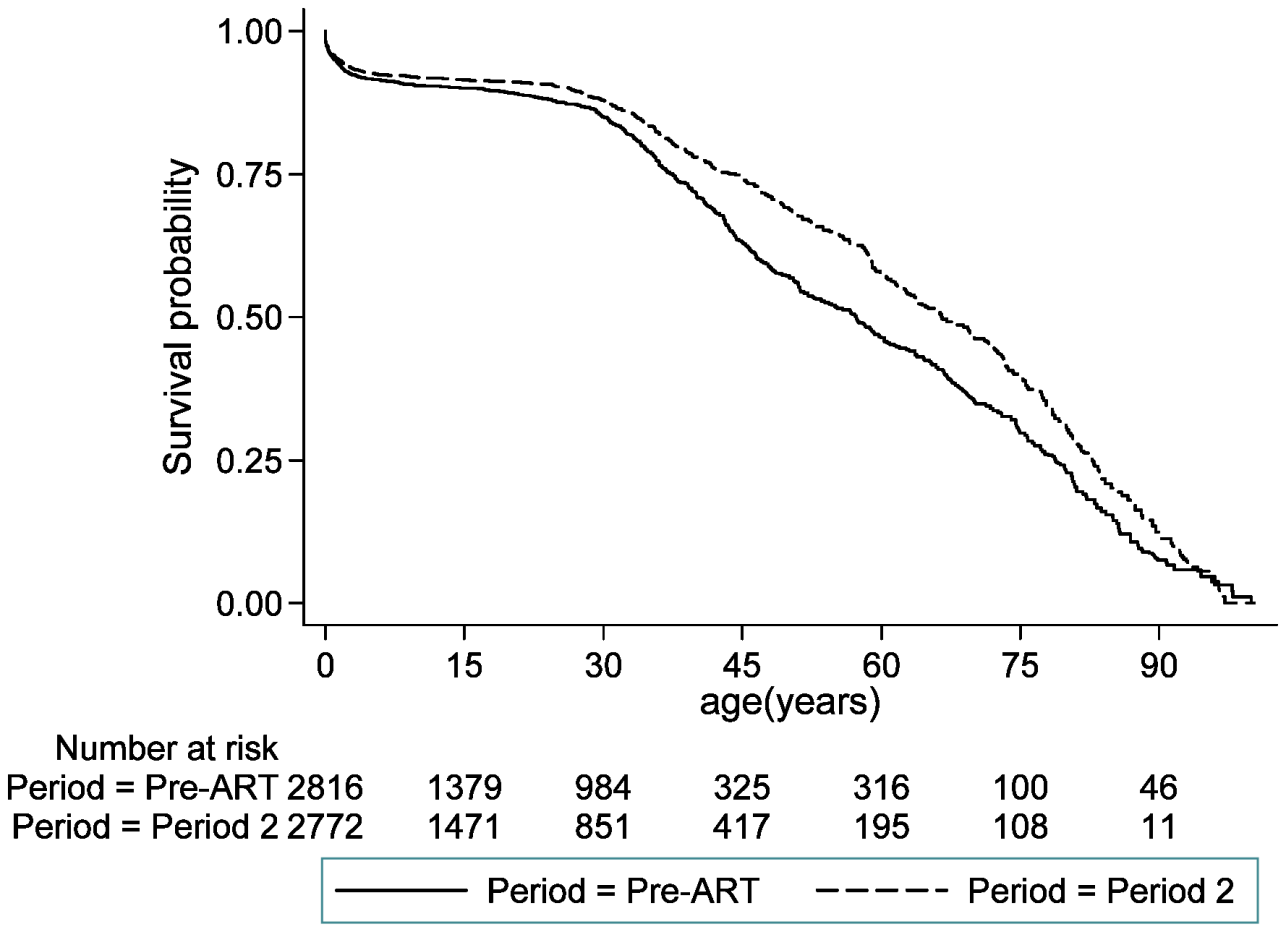
Probability of survival by age in years, separately for the pre-ART period and ART period 2.

Compared with the pre-ART period, the all-cause mortality rate fell by 16% during the time period when ART was available only in the town 70 km from the DSS area (July 2005 to September 2006), and by around one-third in ART period 2 following the opening of a clinic providing ART within the DSS area (p<0.001, Table 1). The all-cause mortality rates were 10.2, 8.5, and 6.9 per 1000 person-years respectively during these three time periods (Table 1). Using the pre-ART and ART period 2 mortality rates to construct two survival curves, the impact of the mortality reduction is seen from around age 25. The probability of surviving to age 60 is 57% in ART period 2 compared with 46% in the pre-ART period (Figure 1).

**Table 1:** All-cause mortality in individuals aged 15–59 years old, by 3 time periods pre-ART and following ART roll-out, overall and stratified by area of residence, sex, and age group.

	Deaths/PYO^1^	Rate/1000 PYO	Rate ratio (crude)	Rate ratio (adjusted)^2^,95% CI and p-value
**Entire study population**				
Pre-ART	288/28285^3^	**10.2**		
ART period 1	158/18514	**8.5**	0.84	0.84 (0.70–1.03), p = 0.09
ART period 2	209/30380	**6.9**	0.68	**0.68 (0.57–0.82), p<0.001**
**By area of residence**				
“Rural remote”				
Pre-ART	93/13476	**6.9**		
ART period 1	73/9596	**7.6**	1.10	1.13 (0.83–1.53), p = 0.45
ART period 2	95/15842	**6.0**	0.87	**0.87 (0.65–1.16), p = 0.34**
“Rural roadside”				
Pre-ART	195/14808	**13.2**		
ART period 1	85/8918	**9.5**	0.72	0.72 (0.56–0.93), p = 0.01
ART period 2	114/14538	**7.8**	0.60	**0.59 (0.47–0.74), p<0.001**
**By sex**				
Men				
Pre-ART	130/13473	**9.6**		
ART period 1	84/8811	**9.5**	0.99	1.00 (0.76–1.32), p = 0.99
ART period 2	102/14407	**7.1**	0.73	**0.73 (0.56–0.95), p = 0.02**
Women				
Pre-ART	158/14812	**10.7**		
ART period 1	74/9703	**7.6**	0.71	0.74 (0.56–0.97), p = 0.03
ART period 2	107/15972	**6.7**	0.63	**0.64 (0.50–0.82), p<0.001**
**By age group**				
15–29 years old				
Pre-ART	59/16395	**3.6**		
ART period 1	31/10601	**2.9**	0.81	0.84 (0.54–1.30), p = 0.44
ART period 2	40/17060	**2.3**	0.65	**0.67 (0.45–1.01), p = 0.06**
30–44 years old				
Pre-ART	147/7870	**18.7**		
ART period 1	79/5384	**14.7**	0.79	0.81 (0.62–1.07), p = 0.13
ART period 2	102/9139	**11.2**	0.60	**0.62 (0.48–0.80), p<0.001**
45–59 years old				
Pre-ART	82/4020	**20.4**		
ART period 1	48/2530	**19.0**	0.93	0.95 (0.67–1.36), p = 0.79
ART period 2	67/4180	**16.0**	0.79	**0.81 (0.58–1.11), p = 0.19**

1 PYO – person-years of observation;

2 Entire study population, adjusted for area of residence, sex and age group; Stratified by area of residence, adjusted for sex and age group; Stratified by sex, adjusted for area of residence and age group; Stratified by age, adjusted for area of residence and sex;

3 these figures differ slightly from those published in 2008, due to minor corrections to the data since then.

The mortality reduction was larger in the “rural roadside” area (from 13.2 in the pre-ART period to 7.8 per 1000 person-years in ART period 2, a fall of 41%) than in the “rural remote” area (where the mortality rate fell from 6.9 to 6.0 per 1000 person-years, a 13% reduction), with statistical evidence that the time trend was different in the two areas (p = 0.04). Reductions in mortality were similar in men and women, though there was weak evidence that the reduction happened earlier among women (p = 0.12). The overall patterns were seen in each of the three age groups, but less strongly in the oldest age group.

### AIDS mortality in 15–59 year olds

AIDS mortality fell dramatically following ART roll-out, by 24% after the first clinic was opened, and by 57% after ART was available within the DSS area, compared with the pre-ART time period (Table 2). The AIDS mortality rates were 6.4, 4.6, and 2.7 per 1000 person-years respectively during these three time periods (Table 2). The large mortality reduction in ART period 2 was seen in both areas, for men and women, and in all age groups. Although the reductions in AIDS mortality were larger in the “rural roadside” area than the “rural remote” area, there was no statistical evidence that the trends were different in the two areas (p = 0.28).There was weak evidence that the reduction in AIDS mortality was larger for women than men (p = 0.07).

**Table 2:** AIDS mortality in individuals aged 15–59 years old, by 3 time periods pre-ART and following ART roll-out, overall and stratified by area of residence, sex, and age group.

	Deaths/PYO^1^	Rate/1000 PYO	Rate ratio (crude)	Rate ratio (adjusted)^2^,95% CI and p-value
**Entire study population**				
Pre-ART	180/28285	**6.4**		
ART period 1	86/18514	**4.6**	0.73	0.76 (0.58–0.98), p = 0.03
ART period 2	81/30380	**2.7**	0.42	**0.43 (0.33–0.56), p<0.001**
**By area of residence**				
“Rural remote”				
Pre-ART	57/13476	**4.2**		
ART period 1	34/9596	**3.5**	0.84	0.87 (0.57–1.33), p = 0.52
ART period 2	37/15842	**2.3**	0.55	**0.56 (0.37–0.85), p = 0.006**
“Rural roadside”				
Pre-ART	123/14808	**8.3**		
ART period 1	52/8918	**5.8**	0.70	0.70 (0.51–0.97), p = 0.03
ART period 2	44/14538	**3.0**	0.36	**0.36 (0.26–0.51), p<0.001**
**By sex**				
Men				
Pre-ART	67/13473	**5.0**		
ART period 1	42/8811	**4.8**	0.96	0.98 (0.67–1.45), p = 0.94
ART period 2	41/14407	**2.8**	0.57	**0.58 (0.39–0.85), p = 0.006**
Women				
Pre-ART	113/14812	**7.6**		
ART period 1	44/9703	**4.5**	0.59	0.62 (0.44–0.88), p = 0.007
ART period 2	40/15972	**2.5**	0.33	**0.34 (0.24–0.49), p<0.001**
**By age group**				
15–29 years old				
Pre-ART	33/16395	**2.0**		
ART period 1	14/10601	**1.3**	0.66	0.69 (0.37–1.29), p = 0.24
ART period 2	8/17060	**0.5**	0.23	**0.25 (0.11–0.53), p<0.001**
30–44 years old				
Pre-ART	102/7870	**13.0**		
ART period 1	48/5384	**8.9**	0.69	0.72 (0.51–1.02), p = 0.06
ART period 2	48/9139	**5.3**	0.41	**0.43 (0.30–0.60), p<0.001**
45–59 years old				
Pre-ART	45/4020	**11.2**		
ART period 1	24/2530	**9.5**	0.85	0.88 (0.54–1.45), p = 0.63
ART period 2	25/4180	**6.0**	0.53	**0.56 (0.34–0.91), p = 0.02**

1 PYO – person-years of observation;

2 Entire study population, adjusted for area of residence, sex and age group; Stratified by area of residence, adjusted for sex and age group; Stratified by sex, adjusted for area of residence and age group; Stratified by age, adjusted for area of residence and sex.

### Non-AIDS mortality in 15–59 year olds

Overall, non-AIDS mortality was slightly higher in the time period following ART roll-out compared with the pre-ART time period (Table 3): the non-AIDS mortality rates were 3.8, 3.9, and 4.2 per 1000 person-years in the pre-ART period, ART period 1 and ART period 2 respectively (Table 3). In the “rural roadside” area, where reductions in AIDS mortality were very large, there was little change in non-AIDS mortality. In the “rural remote” area there was an increase in non-AIDS mortality in both of ART period 1 and period 2 compared with the pre-ART period, but there was no evidence of an increasing trend in non-AIDS mortality *within* the time period following ART roll-out. Among men, non-AIDS mortality changed little over time. Among women, in whom the reduction in AIDS mortality was especially dramatic, there was weak evidence of an increase in mortality that was not attributed to AIDS. There was little change over time for each of the 3 age groups.

**Table 3:** Non-AIDS mortality in individuals aged 15–59 years old, by 3 time periods pre-ART and following ART roll-out, overall and stratified by area of residence, sex, and age group.

	Deaths/PYO^1^	Rate/1000 PYO	Rate ratio (crude)	Rate ratio (adjusted)^2^, 95% CI and p-value
**Entire study population**				
Pre-ART	108/28285	**3.8**		
ART period 1	72/18514	**3.9**	1.02	1.03 (0.76–1.39), p = 0.86
ART period 2	128/30380	**4.2**	1.10	**1.10 (0.85–1.42), p = 0.47**
**By area of residence**				
“Rural remote”				
Pre-ART	36/13476	**2.7**		
ART period 1	39/9596	**4.1**	1.52	1.53 (0.97–2.42), p = 0.07
ART period 2	58/15842	**3.7**	1.37	**1.36 (0.89–2.07), p = 0.15**
“Rural roadside”				
Pre-ART	72/14808	**4.9**		
ART period 1	33/8918	**3.7**	0.76	0.75 (0.50–1.14), p = 0.18
ART period 2	70/14538	**4.8**	0.99	**0.97 (0.70–1.35), p = 0.86**
**By sex**				
Men				
Pre-ART	63/13473	**4.7**		
ART period 1	42/8811	**4.8**	1.02	1.02 (0.69–1.51), p = 0.93
ART period 2	61/14407	**4.2**	0.91	**0.89 (0.63–1.27), p = 0.53**
Women				
Pre-ART	45/14812	**3.0**		
ART period 1	30/9703	**3.1**	1.02	1.04 (0.65–1.65), p = 0.88
ART period 2	67/15972	**4.2**	1.38	**1.39 (0.95**–**2.03), p = 0.09**
**By age group**				
15–29 years old				
Pre-ART	26/16395	**1.6**		
ART period 1	17/10601	**1.6**	1.01	1.03 (0.56–1.90), p = 0.92
ART period 2	32/17060	**1.9**	1.18	**1.20 (0.72–2.02), p = 0.48**
30–44 years old				
Pre-ART	45/7870	**5.7**		
ART period 1	31/5384	**5.8**	1.01	1.02 (0.64–1.61), p = 0.93
ART period 2	54/9139	**5.9**	1.03	**1.05 (0.70–1.56), p = 0.82**
45–59 years old				
Pre-ART	37/4020	**9.2**		
ART period 1	24/2530	**9.5**	1.03	1.03 (0.62–1.73), p = 0.90
ART period 2	42/4180	**10.0**	1.09	**1.09 (0.70–1.70), p = 0.69**

1 PYO – person-years of observation;

2 Entire study population, adjusted for area of residence, sex and age group; Stratified by area of residence, adjusted for sex and age group; Stratified by sex, adjusted for area of residence and age group; Stratified by age, adjusted for area of residence and sex

### ART uptake

Among 14951 individuals aged 15–59 years old who were resident in the DSS area at the time of the 2007/2008 sero-survey, 20.3% (3032/14951) were not found at home or refused to be interviewed, 12.4% (1860/14951) were found and agreed to answer questions (including on previous HIV testing history and prior or current use of ART) but not to give blood for HIV testing, and 67.3% (10059/14951) agreed to answer questions and also gave blood for HIV testing (Figure S1).

Among the 10059 who gave blood for testing, an HIV test result was available for 99.9% (10048/10059), 97.2% were given their HIV test result (9762/10048), and 8.0% (805/10048) had an HIV-positive test result. Twenty-four percent (190/805) reported that they had taken ART, and the Weibull regression model predicted that 31% were in need of ART in 2008 (Table 4), so that uptake as a proportion of those in need was around 77%. The Weibull model was a good fit to the data, illustrated by the fact that the observed median age at death (34.0 years) was close to that predicted by the model (32.0 years). Uptake was higher among women than men and higher among individuals resident in the “rural roadside” than “rural remote” area. Among the 1860 individuals who answered questions but did not give blood for testing on this occasion, 78 were already known to be HIV-positive (from tests conducted during other research studies), and of these 18% (14/78) reported that they had taken ART.

**Table 4:** Self-reported uptake of ART among individuals who gave blood for testing in a sero-survey of all adults aged 15–59 years old who were resident in the DSS area in 2007/2008, and were HIV-positive at the time of testing.

	Number ever on ART/total, and percentage		Percentage estimated to need ART by 2008 – from Weibull regression model
	a/t	%	%
**Overall**	190/805	24	31
**By sex**			
Men	59/285	21	31
Women	131/520	25	31
**By age group**			
15–29	17/183	9	14
30–44	114/442	26	30
45–59	59/180	33	51
**By area of residence**			
“Rural remote”	76/371	20	31
“Rural roadside”	114/434	26	31

Further calculations were done to estimate the total number of individuals on ART in the surveillance area, allowing for non-response. Among all individuals who gave blood for testing, 1.9% (190/10059) reported that they had taken ART, and among all individuals who answered questions but did not give blood for testing, 2.0% (38/1860) reported that they had taken ART. If we assume that ART uptake is 2% among the 20% of individuals who were not found at home or refused to be interviewed, then a further 61 individuals aged 15–59 years old would have been taking ART at the time of the sero-survey, to give an estimated total of 289 individuals taking ART.

Following HIV testing in the sero-survey, all HIV-positive individuals who learnt their status were referred to HIV care services, and 52 initiated ART between end January 2008, when the ART cohort study was initiated, and end September 2008. Among individuals who consented to be interviewed but did not give blood for testing, and among individuals who did not participate in the sero-survey, respectively 4 and 10 individuals started ART during this time. We thus estimate that around 355 (289+52+4+10 = 355) individuals were taking ART by the end of September 2008 (Figure S1). This is an approximate figure: it includes new ART patients without subtracting any who died after they were seen in the sero-survey, but misses those who were on ART but did not report this.

In a sero-survey conducted in 2005/2006 among around 10% of the adult population in the DSS area, 38% of HIV-positive individuals were in need of ART, based on clinical staging and CD4 count data[27]. If we assume an overall HIV prevalence of 10% among the 14951 individuals aged 15–59 years old in the DSS population (allowing for higher HIV prevalence in those not tested in 2007/8, and compatible with the 11.4% found in the 2005/6 survey), and that 34% of HIV-positive individuals are in need of ART (midway between the two estimates of 31% and 38%), then a total of 508 individuals would be in need of ART.

### Survival after starting ART, and mortality prior to starting ART among individuals registered for care at the ART clinic within the DSS area

Among 194 individuals who registered at the ART clinic within the DSS area between January 2008 and June 2009, and started ART, retention in care at 3 months was 94% and at 12 months 91%. Among 112 individuals who were either not eligible, or too sick, to start ART at the time of first screening, 3.6% died and 2.7% were lost to follow-up.

## Discussion

Our findings show that three years after the start of ART clinics in rural, northern Malawi there has been a substantial reduction in all-cause and AIDS mortality among people aged 15–59 years old. For all-cause mortality, our best estimate is that mortality has fallen by one-third during the second and third years of ART roll-out in the district, compared with the pre-ART period, and for AIDS mortality our best estimate is that it has fallen by around 57%. These dramatic reductions have occurred with the adoption of a “public health” approach to ART delivery, which has enabled a rapid increase in treatment coverage and accessibility of care while still achieving good treatment outcomes[10].

It is striking that the overall mortality reduction is much larger since an ART clinic opened within the study area, making treatment more accessible than it was during the first year of ART roll-out. From the start of the third year of ART roll-out in the district, uptake of ART was also enabled by the sero-survey: 65% of adults aged 15–59 years old learnt their HIV status, several hundred were referred to HIV care services, and 52 initiated ART between the end of January and the end of September 2008. The cohort study provision of consistently available CD4 counts at the local clinic may also have enhanced treatment access compared with other areas of Malawi. Even with the opening of the ART clinic in the DSS area, the reduction in all-cause and AIDS mortality remained greater in areas that were closer to the tarmac road, providing additional evidence of the importance of making services as accessible as possible. The relatively low HIV prevalence in the more remote areas also contributes to the lower reduction in all-cause mortality in these areas.

Our estimates of the reduction in all-cause mortality are compatible with estimates of ART need, known levels of ART uptake, and known levels of survival on ART among individuals registered at the clinic that provides ART, in the study area. We have estimated that around 355 individuals were taking ART, with around 508 individuals in need of ART, during the time period covered by the 2007/8 sero-survey. Given that 63% of deaths were attributed to AIDS during the period 2002-5, then if we assume treatment coverage of 70% (355/508), and that the “efficacy” of ART during the first 2–3 years of ART roll-out is to avert 75% of AIDS deaths among individuals on ART (which is broadly compatible with published data on retention in care in Malawi and with data from the clinic in the DSS area), then the proportion of deaths in the population averted by ART provision (the population attributable fraction) would be 33% (i.e. 0.63×0.70×0.75), very similar to the observed reduction.

Other evidence that observed reductions in mortality are due to ART is that there was little change in either non-AIDS mortality or in mortality among individuals aged 60+ years old during the period following ART roll-out. The slight increase in non-AIDS mortality during the period following ART roll-out may be due to misattribution of cause of death. Verbal autopsy reviewers were more likely to have access to information on HIV status in the time period following ART roll-out; as a consequence, the number of HIV-negative individuals whose cause of death was wrongly assigned to AIDS (because they had an AIDS-like clinical condition) will have fallen. Nonetheless, our estimates of 6.4 per 1000 person-years for the pre-ART AIDS mortality rate (corresponding to 6.4 per 100 person-years among HIV-positive individuals if we assume HIV prevalence of 10%), and 3.8 per 1000 person-years for the non-AIDS mortality rate, are broadly in line with published data from Southern and East Africa on the mortality of HIV-positive and HIV-negative individuals[32], [34]. This provides confidence that, at the population-level, verbal autopsies are a useful tool for estimating the proportion of deaths that are due to AIDS.

Our findings are also in line with other studies of the population-level impact of ART on AIDS mortality in the early years of ART roll-out. The estimated 57% (95% CI 44–67%) reduction in AIDS mortality with ART coverage of around 70% found in our study is consistent with a 50% reduction in Addis Ababa during 2 years in which ART uptake was apparently very high[21] as well as the approximately 25% reduction in a rural area of South Africa with ART uptake averaging around 40% of those in need during the first 3 years of ART roll-out[22], and a 37% fall in registered deaths in a district of southern Malawi with treatment coverage estimated to be around 80%[24]. Overall, our findings support the roll-out of ART treatment to health centres and clinics with unsophisticated facilities: we believe that increased access to ART is the primary reason for the sustained fall in all-cause and AIDS mortality, although the sero-survey conducted in 2007/8 made a contribution and other temporal changes in health-seeking behaviour might also have made a difference.

A key question is whether the reduction in all-cause and AIDS mortality can be sustained or even increased. ART coverage in the study area is already around 70% using current treatment criteria in Malawi, but recently adjusted criteria will see the numbers of HIV-infected adults who are eligible for ART increase[35], while increased access to HIV testing and increased confidence in the health care system will enlarge the numbers seeking care. Set against this, enhancing access may be constrained because many clinics in Malawi are already operating at high capacity[10], and further task-shifting will probably be required[36]. Roll-out of ART to the next layer of health clinics and health posts seems justified in order to further increase access and limit the impact of the ART programme on hospital facilities, whilst increasing the capacity to manage the rising number of HIV-infected individuals who need treatment. Localising care is also expected to enhance adherence to therapy and retention in treatment programmes.

It has been recognised that the early years of ART roll-out are a “honeymoon” period during which it is possible to avert a high proportion of AIDS deaths[10]. Dramatic mortality reductions at the population level can be sustained only if the survival of individuals on ART is prolonged for as long as possible, rather than for only a few years, and if HIV prevention measures are implemented at the same time since HIV prevalence will increase due to ART provision unless HIV incidence simultaneously falls.

In conclusion, our study shows that ART can have a dramatic effect on mortality in a resource-constrained setting in Africa, at least in the early years of treatment provision. Continued monitoring of this effect is essential, to confirm if it can be sustained and to anticipate and plan changes to continue this successful intervention. Continued funding to maintain and further scale-up treatment provision will bring large benefits in terms of saving lives.

## Supporting Information

Figure S1 Uptake of HIV testing in a sero-survey conducted 2007/8, and reported and subsequent uptake of ART, among individuals aged 15–59 years old.
(0.01 MB PDF)
