# Supplementary figures and images for: Population-Level Reduction in Adult Mortality after Extension of Free Anti-Retroviral Therapy Provision into Rural Areas in Northern Malawi
Source: PLoS One. 2010 Oct 19;5(10):e13499. doi: 10.1371/journal.pone.0013499 (PMC2957442; doi:10.1371/journal.pone.0013499)

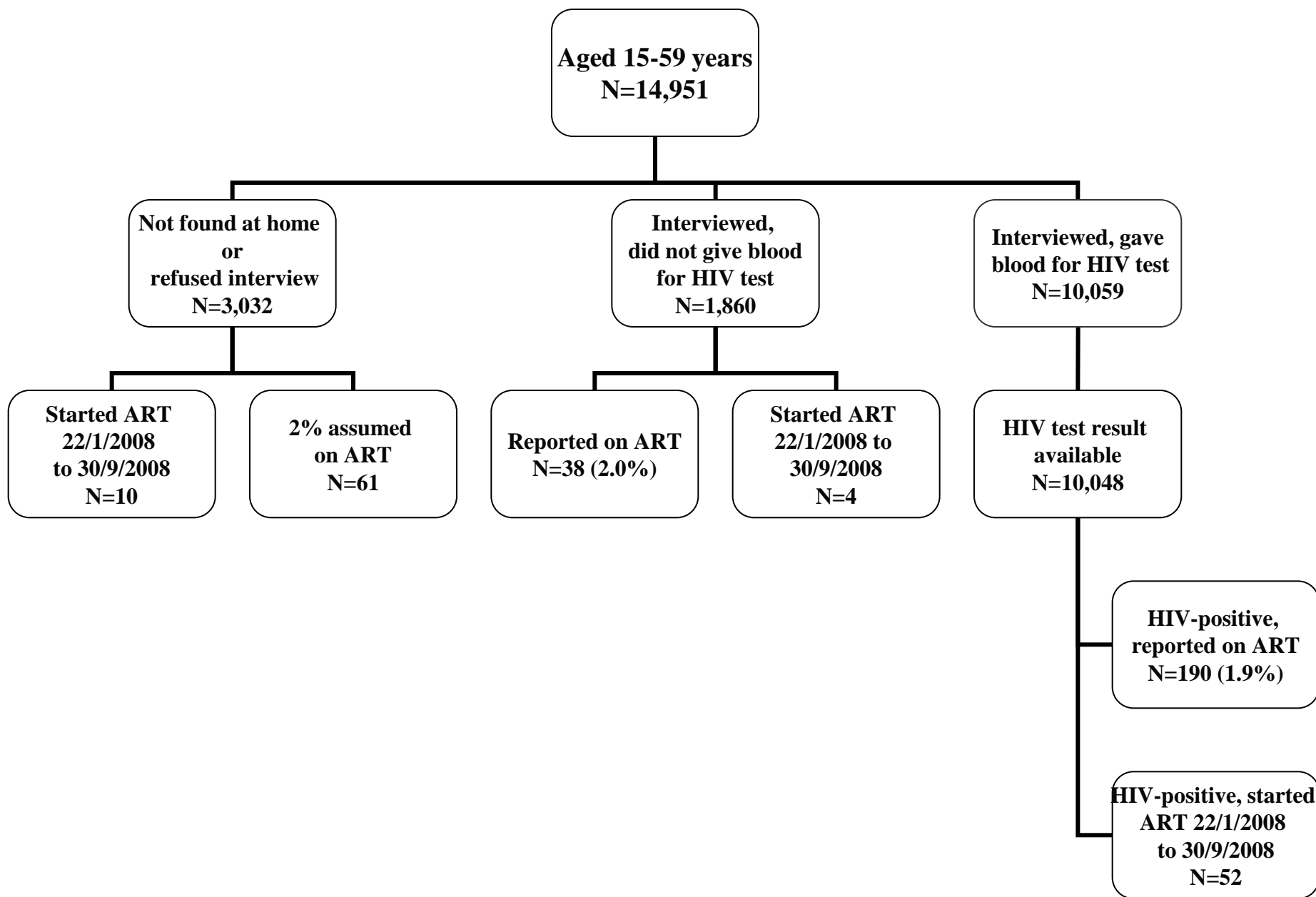

Supplement: Figure S1 — Uptake of HIV testing in a sero-survey conducted 2007/8, and reported and subsequent uptake of ART, among individuals aged 15–59 years old. (0.01 MB PDF) [file pone.0013499.s001.pdf]
